# Developing guidance for the management of intraoperative anaesthetic machine failure

**DOI:** 10.1177/17504589221082851

**Published:** 2022-03-17

**Authors:** Benjamin Milne, Kate Prior

**Affiliations:** 1 Department of Anaesthesia & Pain Medicine, King’s College Hospital NHS Foundation Trust, London, UK; 2 King’s College London, London, UK; 3 King’s College Hospital NHS Foundation Trust, London, UK

**Keywords:** Anaesthesia, Anaesthetic machine, Equipment failure, Emergency guidance, Critical Incidents

## Abstract

Intraoperative anaesthetic machine failure represents an immediate risk to patient safety, as well as risking long-term harm in the form of accidental awareness under general anaesthesia. Currently, there is no widely accepted consensus guidance for the management of such an event. Based upon institutional experiences of anaesthetic machine failure and the principles of delivering good-quality care under emergency scenarios, we devised a single-sheet guideline for management of this event. This guidance assigns clear roles in the management of the event, identifies the key priorities for immediate care, and makes provisions for ensuring ongoing high-quality care following the event. Discussion is given to the rationale for the key components, and the importance of involving the whole perioperative team in developing such guidance. Further discussion involves the crucial elements of local implementation, making sure that guidance is location and personnel specific. Key future steps in this important patient safety project are also discussed.

## Introduction

Intraoperative anaesthetic machine failure is an emergency requiring urgent management to avoid serious risk of harm to the patient, such as hypoxaemia and accidental awareness under general anaesthesia. Machine failure can represent dysfunction of the entire machine, or of the constituent elements, including failure of anaesthesia delivery (via the vaporisers), failure of oxygen or other gas delivery, and failure of the mechanical ventilator or incorporated monitoring devices. In the emergency event of machine failure once a patient is anaesthetised, and during surgery, identification of the exact site and nature of the fault is a secondary priority after ensuring the safety of the patient, by maintaining oxygen delivery and alternative anaesthesia. However, consideration of these technical elements will need to be incorporated into any ongoing management plan.

Incidence of machine failure is difficult to ascertain, certainly in terms of index cases per anaesthetic. One registry study of patient safety reports found 1029 cases of anaesthetic equipment issues over a two-year reporting period. Of these, 26.4% involved monitoring issues (8.9% were failure of monitoring during anaesthesia), 17.9% were ventilator problems (including sudden failure during anaesthesia in 13.8%), with other reported issues including dysfunction of gas monitoring (13.4%), vaporiser problems (5.1%) and alarm failure (1.4%). Approximately 10% of these incidents were felt to involve harm to the patient (Cassidy et al 2011). Under-reporting is almost certain to have occurred in this cohort (Smith et al 2006) and another study has suggested that the anaesthetic machine was the commonest cause of all equipment problems in anaesthesia (31%) (Fasting & Gisvold 2002). Establishing a true denominator for these cases has thus far proved elusive.

Anaesthetic machine failure, although seemingly relatively uncommon, is a reasonable cause for concern for both practitioners and patients alike. As such the Royal College of Anaesthetists (RCoA 2017) provides written information for patients concerning how such an event would be managed. However, there is no consensus guidance for management of intraoperative anaesthetic machine failure within the United Kingdom, despite the extensive arsenal of single-sheet guidelines available for the management of emergencies in anaesthesia, much of which is provided by the Association of Anaesthetists. A potential reason for the absence of such guidance may be due to the consideration that management of crises involving ventilation and hypnotic provision is such an integral element of the core skill set of anaesthetists, that written guidance is unnecessary. However, there are several key issues to consider regarding the aptness of guideline development.

First, it is important to consider that anaesthesia delivery in the United Kingdom is undertaken by anaesthetists of varying grade and experience, a cohort which includes anaesthesia associates, resulting in a broad spectrum of clinical practice. They may be working under indirect supervision from a consultant anaesthetist, and supported by anaesthetic nurses and operating department practitioners (ODPs), who can be an invaluable source of experience and knowledge. Furthermore, practitioners are providing anaesthesia in more diverse clinical environments, and for cases with increasing patient and procedure-related complexity. Developing a broad armoury of guidelines for routine standards of care will help reduce cognitive burden during the ongoing expansion of practice. In this respect, guidelines are firmly adjunctive to clinical knowledge and expertise and not a replacement (Goldhaber-Fiebert & Macrae 2018, Simmons & Huang 2019).

Second, there is the consideration as to the purpose of developing clinical guidelines. Guidance offers a framework for the safe management of a condition or scenario, and while the expert may choose to deploy their considerable knowledge and skill in doing so, for more junior anaesthetists the presented framework may be more prescriptive, and, in parallel, more useful. While all anaesthetists undertaking practice with anything less than full direct consultant supervision will be trained and expected to manage instances of intraoperative machine failure, there is evidence for variability in ability in management of such an event. One simulation-based study demonstrated that across a cohort of anaesthesia trainees, seniority played a part in successful management of equipment failure, however even when the training year was taken into consideration, there was significant variability in performance (Waldrop et al 2009). Establishing guidelines and local protocols is one such way of reducing variability in performance, and, therefore, reducing disparity in the quality of care that patients receive. Standardisation of practice is an important tool in improving patient safety and care, and while adoption of items such as critical event checklists and emergency manuals can be variable, their use is increasing and has been associated with better performance of trainees in the operating theatre (Siddiqui et al 2019), and are of particular value in the management of less routinely encountered scenarios (Hepner et al 2017, Simmons & Huang 2019).

Finally, the process of developing guidelines encourages critical assessment of local resources and serves as a point of agreement between several different teams, both clinical and non-clinical, as to how an event is to be managed. Unlike some emergencies in perioperative anaesthesia care where management solely involves the anaesthetist and their trained assistant, anaesthetic machine failure has roles for numerous members of the perioperative team and will be more effectively managed by planning for the inclusion of these members. Furthermore, an agreed management strategy provides a tool to highlight to support staff, for example, those involved in equipment management, what equipment is required and to be maintained in all theatre environments.

Given this rationale, and after a local occurrence of machine failure, when the logistic issues of providing alternative anaesthesia, ventilation and monitoring needed to be considered, we decided to develop local guidance for the management of such situations.

## Discussion

**Figure 1:**
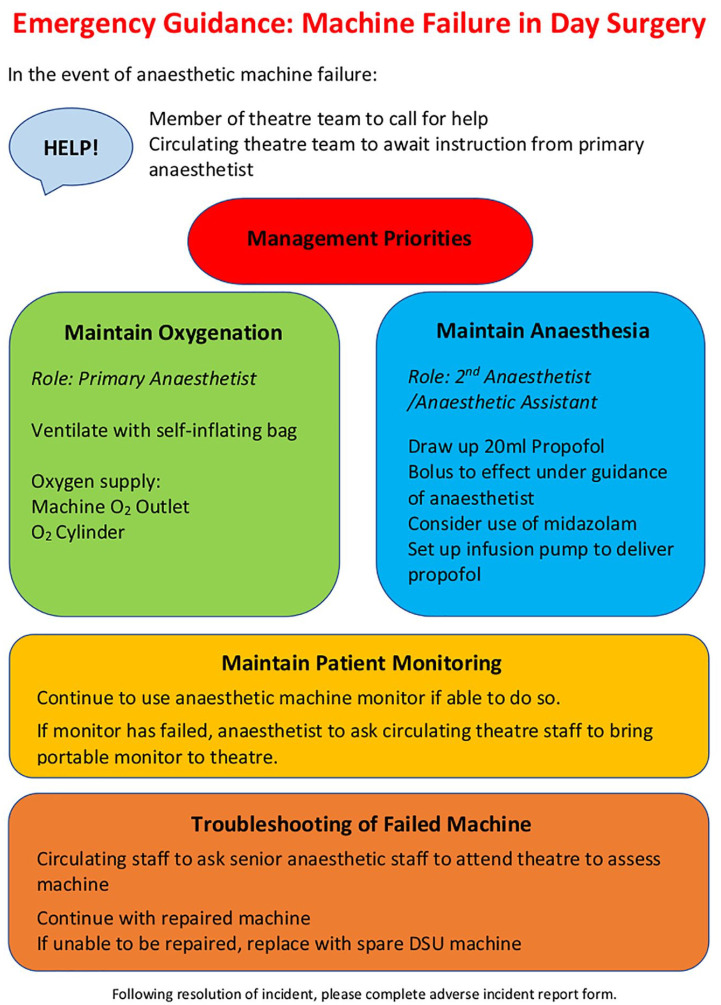
Single-sheet guidance for management of intraoperative anaesthetic machine failure

We produced a single-sheet guideline for the management of intraoperative anaesthetic machine failure (Figure 1). The guidance deals with the immediate priorities of management, namely hypoxaemia and accidental awareness, while ensuring high-quality ongoing patient care.

### Scope and purpose

We aimed to develop guidance for the management of intraoperative machine failure, based upon a prepared team-based response, for use within our large teaching hospital. Within our institution, 27 operating theatres are in routine use, across a broad range of surgical specialties, as well as six specialty procedure areas, such as endoscopy and interventional radiology suites, where anaesthesia is regularly required. Given the scope of diversity in specific areas, we initially developed our guidance for our day surgical unit, which houses seven operating theatres, catering to a broad range of surgical specialties, including paediatric subspecialties.

Furthermore, as a teaching hospital there is a broad spectrum of experience within the perioperative team, supervised by experienced clinical staff. Within the anaesthetic team, consultant anaesthetists work with trainees from their novice period at the start of training, through to senior subspecialty training fellows. Similarly, experienced operating department practitioners and anaesthetic nurses work alongside more junior practitioners. Our guideline considers all levels of experience and is aimed at all practitioners.

### Guideline development

The guideline was developed using a multi-disciplinary approach, with input from the wider perioperative team. Anaesthetic departmental experience of machine failure was sought, and an appropriate clinical resolution was developed from insight provided by anaesthetists, anaesthetic nurses and ODPs. Consideration was given to local reports of machine failure, including the issues that arose, and the strengths and weaknesses of the management strategies employed, as well as the evidence in the subsequent section.

The key aspect in development was considering the concerns of the wider operating department team. While the clinical situation faced will often be straightforward, and management should be improved by provision of a simple protocol, institutional experience has demonstrated that there may be inefficiencies or shortcomings related to wider staff utilisation and availability of equipment. By incorporating the perspective and experience of other members of the theatre team, we have produced a guideline which serves as an agreement between all involved parties, both clinical and non-clinical, as to how such situations should be managed, which members of staff should be familiar with the location of certain equipment, and correspondingly what equipment should be maintained in certain areas.

Internal validation was sought from the relevant team leaders, including the anaesthesia clinical lead for day surgery, lead for ODP/anaesthesia nursing and lead for technical and equipment support staff. Given the specificity of the guidance to the clinical area, subsequent implementation in other areas will require input and validation from those relevant stakeholders.

### Content and rationale

The guideline developed follows a chronological order and clearly prioritises the most important aspects of clinical care. Furthermore, the structure of the guidance should appear familiar to perioperative practitioners. Crisis Resource Management (CRM) principles have become well ingrained into anaesthetic practice, and our management guidance sits well within this framework (Rall & Gaba 2009). Calling for help early is a key tenet of these principles, and in line with the management of all anaesthesia-based critical incidents, this is highlighted in the guideline. While the anaesthetist and the ODP or anaesthetic nurse will be focused upon the patient and their management, our guidance encourages a member of the wider theatre team to call for help, while circulating team members should be prepared to take instruction from the principal anaesthetist. Role assignment is crucial, and our guidance formalises this measure, and gives agency to staff for self-assignment. Theatre staff, including ODPs, anaesthetic and surgical nurses, have familiarity with a wide range of perioperative equipment, and with their working environment, which will enable them to facilitate provision of care in a time-critical fashion. The guidance should empower them to take the lead in seeking the required alternative equipment, and locate appropriate support from theatre equipment technicians or experienced ODPs/anaesthetic nurses.

Once the team has been alerted to the ongoing emergency, the guideline follows CRM principles, for the effective and efficient use of both time and personnel, allowing for appropriate distribution of workload, mobilisation of all appropriate resources, while setting management priorities, and giving clear assignment of roles. Furthermore, the tool is itself the cognitive aid that CRM principles would recommend (Rall & Gaba 2009).

For maintenance of oxygenation, the most crucial priority, we have advised use of a self-inflating bag. While other alternatives are available, for example, a Mapleson C circuit, a self-inflating bag has the benefit of being able to ventilate the patient even in the absence of an oxygen source, and availability should be ensured as guidance recommends this equipment should be present wherever anaesthesia is conducted (AAGBI et al 2012). A second anaesthetist should be prepared to draw up and administer propofol, or an ODP/anaesthetic nurse under instruction, depending upon hospital policy relating to medicine management in an emergency. It is their familiarity with the drug, and drug administration in general, that means this should be maintained within the anaesthetic team as much as possible, which is an important safety step. Similarly, the secondary anaesthetist’s familiarity with infusion pumps makes them the most appropriate to set these up, assisted by the anaesthetic nurse/ODP who will be familiar with their function.

We have advised the consideration of midazolam use, although this issue may prove contentious. Administration of midazolam produces anterograde amnesia, but does not appear to produce, even immediate, retrograde amnesia (Bulach et al 2005). Therefore, while midazolam administration may not prevent episodes of accidental awareness from an established failure in the delivery of anaesthetic agent, it may provide protection against further anaesthesia insufficiency during emergency administration of propofol by manual bolus or attainment of adequate plasma concentration during institution of infusion. This may be of particular importance if a target-controlled infusion of propofol is to be used, as this will start from an unusual baseline (ie: an already anaesthetised patient), where the effect site or plasma target level may be set relatively conservatively by the anaesthetist, to avoid haemodynamic instability. Midazolam administration may be counterproductive to the aims of day surgery anaesthesia, which is where our guideline has first been implemented, although this is contested. However, if the aim is to reduce accidental awareness, it is worthy of consideration (Gurunathan et al 2020, Hyunjee et al 2020).

Maintenance of adequate patient monitoring is a crucial responsibility (Klein et al 2021), and if able to do so, the main monitor should be used. If not, a portable monitor should be available, and be retrieved by staff members, who should be familiar with its location.

The above steps ensure the key priorities of maintaining anaesthesia and ventilation, while ensuring the patient is adequately monitored. These steps may be sufficient should the surgery be close to conclusion, however, prolonged use of a self-inflating bag is tiring for the operator, does not permit accurate incrementation of inspired fraction of oxygen, and risks hyper or hypoventilation. Therefore, our guidance advocates that while the clinical staff continue to deal with the patient, a technical solution should be sought for the failed machine. If a repair is possible and timely, this can be performed, otherwise a spare machine should be brought into the theatre suite, or an alternative local arrangement as agreed, which could include the use of a portable or transport ventilator.

The increasing availability of dedicated technical support in the theatre environment, including high-quality auxiliary equipment, has permitted emphasis on the concept of introducing alternative machine/devices to ensure that quality of patient care does not deteriorate following such an incident. For the initial implementation of our guideline, an area was chosen with full-time technical support from a clinical engineer from the Electrical and Biomedical Engineering (EBME) Department within the theatre suite (along with a designated spare anaesthetic machine), which permits our agreed protocol of a technician attending and supporting rapid troubleshooting and resolution of machine dysfunction, or assisting with replacement. This simultaneous approach to patient and machine management allows the technical skills of all team members to be most appropriately employed, particularly as anaesthetists may underperform when identifying faulty equipment (Larson et al 2007). However, dedicated technical support such as this is not universal, and can differ by Trust, hospital, and even by clinical area and time of day. For expansion into other clinical areas, and to maintain round the clock applicability, this role should change to an appropriate alternative member of staff, while the duty of this role should change to accommodate the person performing it, ie: an in-hours technician trouble-shooting role, may be more appropriately substituted for an ODP/anaesthetic nurse retrieving alternative devices to replace malfunctioning equipment out of hours. For other hospitals implementing similar guidance, the potential absence of such widely available equipment technicians should change the exact role assignment, but does not reduce, and may increase, the importance of this individual task.

### Implementation

For the initial implementation of our guidance, we chose day surgery, an area where surgery is performed within normal working hours, thus with higher levels of 1:1 consultant anaesthetist to trainee supervision. Implementation should be a joint effort of the whole perioperative team, including anaesthetists, ODPs and anaesthetic nurses, specialist theatre nursing staff, and clinical engineering staff. Successful implementation of any patient safety intervention must account for the skill and competencies of the individual, effective team-working and the clinical environment, all of which are possible with this guidance (Sevdalis et al 2012). The integration into clinical practice can also further foster a culture of teamworking and open communication (Neily et al 2010, Simmons & Huang 2019).

At our institution, we have implemented the use of laminated guideline posters, within the anaesthetic area of each theatre, adjacent to the anaesthetic machine, and have ensured awareness of the entire perioperative team to both presence and purpose. Further processes of implementation are made possible with this guideline, including in situ simulation training, which has become a vital part of health care education. Anaesthetic machine failure is an uncommon occurrence, requiring deft handling to prevent poor patient outcomes, suggesting it is an appropriate area for simulation training. One simulation-based study has demonstrated that while management of equipment failure-related scenarios improved with increasing trainee seniority, it then reached a plateau, potentially relating to lack of additional training opportunities, and the relative infrequency of failure in practice. While not all trainees will experience the same opportunities to manage all real-life intraoperative emergencies, simulation can ensure that there is homogeneity in skill acquisition and maintenance (Waldrop et al 2009). Our guidance forms a template for in situ simulation, involving the whole theatre team, and would provide a robust approach to integration into clinical practice (Sevdalis et al 2012).

### Quality improvement

Quality Improvement aims to improve safety, effectiveness and patient experience of care, using a systematic approach, with design, testing and implementation of change by an appropriate real-time measure of improvement (Jones et al 2019). Intraoperative anaesthetic machine failure is a rare occurrence, which has implications for use of a standard quality improvement methodology for this project. The low incidence will mean there is likely to be a long period between cases for which alterations can be made to the guideline, impairing real-time measurement of improvement. Considering this, an alternative methodology for review and ongoing improvement has been devised. The guideline encourages reporting of adverse incidents, in line with national policies, and these reports will be monitored to enable a process of feedback regarding guideline performance and improvement, using a targeted questionnaire, considering the guideline and team performance. Finally, this patient safety project will be extended to other theatre areas, adapting to reflect the resources and personnel which are available in those areas in and out of hours, incorporating the feedback emerging from the initial implementation.

### Strengths and limitations

We have produced a simple guideline for the management of intraoperative machine failure, which identifies the key roles and responsibilities of theatre practitioners, and uses the available resources to produce a rapid, team-based response. The main limitation of our guidance is that it is not immediately generalisable to other areas or sites, and, by nature of its specificity, may not be uniform across all clinical areas on our clinical site. We have emphasised that in order for our guidance to be safe and effective, it should reflect the specific resources that are guaranteed to be available in the chosen clinical area. A balance has been struck between ensuring consistency across a site, with making sure the proposed guidance is appropriate to the clinical area, and the patient group being managed there. The key to successful adaptation and implementation in other clinical areas, and therefore at other hospitals, will be in making the guidance location and personnel-specific, accounting for local resources, and removing areas of uncertainty which may hinder team performance. Therefore, our guideline would not be appropriate for immediate implementation elsewhere, but could serve as a framework for developing local guidance.

## Conclusion

We have developed guidance for the management of intraoperative anaesthetic machine failure. The guidance is aimed to standardise practice, particularly for less experienced anaesthesia staff, and to ensure ongoing high-quality patient care in the immediate aftermath of machine failure, but also following initial management. Our guidance is specific, not just to our Trust, but to the specific clinical environment for which it was intended. We would encourage other departments to develop similar guidance, considering their own specific resources and clinical environments.
